# CYP1B1-mediated Pathobiology of Primary Congenital Glaucoma

**DOI:** 10.5005/jp-journals-10008-1189

**Published:** 2016-02-02

**Authors:** Muneeb A Faiq, Rima Dada, Rizwana Qadri, Tanuj Dada

**Affiliations:** Student, Dr Rajendra Prasad Centre for Ophthalmic Sciences, All India Institute of Medical Sciences, New Delhi, India; Professor, Laboratory for Molecular Reproduction and Genetics Department of Anatomy, All India Institute of Medical Sciences New Delhi, India; Student, Department of Laboratory Medicine, All India Institute of Medical Sciences, New Delhi, India; Professor, Dr Rajendra Prasad Centre for Ophthalmic Sciences, All India Institute of Medical Sciences, New Delhi, India

**Keywords:** CYP1B1 gene, Functional genomics, Glaucoma, Intraocular pressure, Pathobiology, Primary congenital glaucoma, Trabecular meshwork.

## Abstract

CYP1B1 is a dioxin-inducible enzyme belonging to the cytochrome P450 superfamily. It has been observed to be important in a variety of developmental processes including *in utero* development of ocular structures. Owing to its role in the developmental biology of eye, its dysfunction can lead to ocular developmental defects. This has been found to be true and CYP1B1 mutations have been observed in a majority of primary congenital glaucoma (PCG) patients from all over the globe. Primary congenital glaucoma is an irreversibly blinding childhood disorder (onset at birth or early infancy) typified by anomalous development of trabecular meshwork (TM). How CYP1B1 causes PCG is not known; however, some basic investigations have been reported. Understanding the CYP1B1 mediated etiopathomechanism of PCG is very important to identify targets for therapy and preventive management. In this perspective, we will make an effort to reconstruct the pathomechanism of PCG in the light of already reported information about the disease and the CYP1B1 gene.

**How to cite this article:** Faiq MA, Dada R, Qadri R, Dada T. CYP1 B1-mediated Pathobiology of Primary Congenital Glaucoma. J Curr Glaucoma Pract 2015;9(3):77-80.

## CYP1B1 AND OCULAR DEVELOPMENT

CYP1B1 is an important gene for developmental orchestra and plays ardent role in the embryonic development. It is a developmentally important gene having indispensible roles to play explicitly in ocular developmental biology. Important studies have reported the presence of CYP1B1 mRNA in a variety of fetal tissues^1^ and anatomical compartments―most importantly kidneys. This is to mention that these studies were silent about many details of the samples used to detect CYP1B1 mRNA in the fetal tissues. RT-PCR based studies on CYP1B1 expression have reported its constitutive expression in many fetal tissues, including brain, adrenal glands, liver and kidneys in the first and second trimesters.^2^ The expression of this gene almost ubiquitously in fetal tissues and anatomical compartments implies its importance in development and advocates its involvement in developmentally important disease which includes primary congenital glaucoma (PCG). Mutations in CYP1B1 gene or any other derangement precipitating its functional deficit, by this logic, may cause congenital malformations which include PCG^3,4^ and, by extension of this logic, other anterior segment disorders. What is important to note is that CYP1B1 null mice are, as such, normal^5^ but peculiarly display isolated defects in tissues relevant to PCG.^6^ Additionally, CYP1B1 has been found to be important in pregnancy maintenance because CYP1B1 polymorphisms are observed to be imperative in proper functioning of estrogen and progesterone receptors.^7^ A relevant point to note here is that V432L variant of the wild type has quadrupled K_m_ for stereospecific 4-hydroxylation of 17p-estradiol which may be mediated through a drift in estradiol binding affinity.^8^

## PRIMARY CONGENITAL GLAUCOMA GENETICS AND CYP1B1

Primary congenital glaucoma, a blinding disorder with onset at birth or during infancy, mainly presents with developmental abnormality in trabecular meshwork (TM) often coupled with other associated symptoms. Trabecular meshwork dysgenesis causes an obvious obstruction to the aqueous outflow precipitating increase in the IOP leading to ocular hypertension mediated optic nerve cupping and consequent loss of vision.^9^ Primary congenital glaucoma has autosomal recessive mode of transmission with varied prevalence (1:10,000 in the western world to 1:1,250 among the Slovak Gypsies).^10^ Consanguinity has been found to be a fundamental mechanism for increased PCG prevalence in certain population like Saudi Arabians and Slovakian Gypsies. Primary congenital glaucoma is a complex disease which has been mapped to at least three genetic loci *viz* GLC3A (GLC abbreviates to glaucoma, three means the congenital form of glaucoma and the suffix A, B and C reveals the chronological order of the deciphering of these loci) chromosome 2 (2p21);^11^ GLC3B―chromosome 1 (1p36);^12^ and GLC3C―chromosome 14 (14q24.3).^13^ The majority of PCG cases present with GLC3A involvement, specifically pointing toward CYP1B1 gene-based etiology.

## CYP1B1 AND PCG MECHANISM

CYP1B1 is pivotal in the proper development of TM (the most important tissue with regards to PCG)^14,15^ and also in the *in utero* development of many other ocular tissues.^16^ The exact mechanism by which CYP1B1 leads to proper TM development is not known but its importance in development and maintenance of TM has well been opined. One important domain of our ongoing work is focused on this aspect of PCG pathogenesis. Some investigators hypothesize that it is either the metabolic conglomeration of some important unknown metabolite (indispensible for ocular development) or the elimination of some, yet unidentified, toxin (detrimental to proper eye development) that may be the mechanistic instrument of CYP1B1-mediated TM dysgenesis in PCG. However, a third mechanism can also be thought of in which CYP1B1 may be orchestrating the expression of some important genes relevant to anterior chamber formation.^14,15^ Mutations detrimental to the CYP1B1 function can, therefore, be thought of as critically pivotal in deranging the proper development of TM and subsequent reduction in aqueous outflow and consequent elevation of IOP. More than 140 mutations in CYP1B1 gene have been reported in PCG cases from all over the world till date^17,18^ many of which have been exclusively reported to be present in PCG cases.^4,14,15,19-27^ Panicker et al recently reported eight mutations in CYP1B1 gene in Indian population many of which were present in the conserved regions and the functionally important regions (FIRs).^28,29^ We have also reported similar results from North Indian population.^30,31^ The complete mechanism of CYP1B1-mediated PCG pathogenesis has not been worked out but it has been proved beyond any reasonable doubt that CYP1B1 is one of the main genes involved in etiopathogenesis of PCG. Elucidation of the etiopathobiology of PCG is a severe need of the hour and can be done through functional characterization of the mutations that have been exclusively reported in PCG. Once the functional characterization of these mutations is accomplished, the vital molecular nodes in pertinent pathways can be identified and worked out which may lead to important interventions for effective treatments. For this reason, functional characterization of novel CYP1B1 mutations found in CYP1B1 cases is imperative and stands justified with a clinically bolstered rationale.^32^

## FUNCTIONAL INSIGHTS

There are a number of ways to execute the functional characterization of these mutations but the most viable would be to express the wild and mutant types in bacterial expression systems, purify them and then compare their activities with respect to various metabolic processes including, but not limited to, estradiol metabolism, retinoid metabolism, arachidonate metabolism and melatonin metabolism (pathways crucially imperative in eye development). For such studies, heterologous expression of unmodified, full length human CYP1B1 is necessary. Our research group has already reported an easy and novel protocol for the heterologous expression of unmodified, full length human CYP1B1 in *E. coli^33^*Structural modeling should also be done to supplement such *in vitro* studies as they yield some important information that may prove pivotal in deciphering the mechanism of function loss in mutants in comparison to wild type of CYP1B1. One good news in this regard is that the crystal structure of CYP1B1 (507 amino acid stretch) has been recently reported. Its PDB ID is 3PMO [assessable at: http://www.rcsb.org/pdb/explore.do?structureId=3pm0]. Structure can be modeled using many softwares like MODELLER [assessable at: https://salilab.org/modeller/download_installation.html]. The pathogenecity of each mutant can also be predicted by in silico methods like PolyPhen (Polymorphism Phenotyping) [assessable at: http://genetics.bwh.harvard.edu/pph2/] and SIFT (Sorting Intolerant from Tolerant) [assessable at: http://sift.jcvi.org/] analyzes. Not only this, stability of the mutant protein as compared to the wild type can also be checked with in silico methods. Many softwares are available for this purpose (e.g. SNAP2 and I-Mutant, etc.). Functional studies are important because the disease causing mutations are mostly present in the functionally important regions (FIRs). We have recently reviewed the mutational update of the CYP1B1 gene in PCG and reported classified mutations exon-wise, country-wise, population-wise and with respect to many other parameters.^18^ As is the generally accepted notion, disease causing mutations in CYP1B1 gene actually cause derangements in the protein structure in the mutant forms ensuing functional insufficiency. This means that functional characteristics of wild-type protein and its mutant forms are different; this causes alterations in enzymatic activity (in the mutants) with respect to various substrates^34^ leading to physiological anomalies and precipitating disease phenotype.

## FROM MUTATION TO LOSS OF FUNCTION

Mutations at important positions are accompanied with the loss of function in the protein product. This is the conceptual basis of the mutational studies that are being carried out for various diseases. Most important mutations occur in the areas that correspond to the FIRs, like substrate binding sites, substrate access channels, heme binding regions, etc. A simple speculation for the probable mechanism is that a mutation leads to changes in the protein structure due to substitution, addition or removal of one or more amino acids and the ensuing change in charge distribution and other significant properties. Once one type of amino acid is substituted by the other (or other alterations arise), many changes occur *viz* surface charge distribution, protein stability, protein flexibility, folding patterns, cell trafficking, core region packing, hydrophobic interactions, catalytic site shape, substrate binding region, substrate access channel, metal binding efficiency, etc. So, an amino acid change brings in many alterations and the nature of change depends on the nature and position of the amino acid altered. Some mutations, for this reason, are well tolerated while others are not.^35-37^

Some investigators have devised a few important points to consider for quality assessment of the extent of deleterious nature of a mutation.^38,39^ The genotype-phenotype correlation studies reported by Panicker et al^40^ adjudge some mutations (they found in their population cohort) deleterious for protein function. This led them to report quantitative measure of severe phenotypes in the Indian population with regards to these mutations [P193L (62.5%), E229K (80%) and R368H (72%)]. An important phenomenon that warrants attention is that most of PCG cases with CYP1B1 mutations present with incomplete penetrance making the understanding of the disease mechanism and mode of inheritance intricately obscure.^25^ This observation bolsters the endeavors for functional characterization of CYP1B1 specific mutations and the characterization of FIRs. An important application of such studies is that they will aid in revealing the etiopathomechanism of PCG and will help in identifying the potential molecular targets for therapy and preventive management.

## CONCLUSION

Mutations in CYP1B1 gene are the major cause of PCG. Many mutations in CYP1B1 gene have been reported in PCG patients. A proper understanding of how these mutations lead to disease phenotype is necessary. This may help in understanding the disease and devising necessary steps to curb it. Functional characterization and molecular modeling studies provide a good hope for understanding the CYP1B1-mediated pathogenic mechanism of PCG. The whole premise works on the considerations that CYP1B1 is important in ocular development, its mutations lead to malfunction which cause maldevelopment of ocular structures leading to PCG. Mutations affect protein structure and function. Expressing the gene and its relevant mutants in heterologous hosts and subjecting to various tests is likely to yield important information that may prove essential in development of novel treatments for PCG.
